# Perception de l'allaitement maternel et de la diversification alimentaire dans une zone urbaine congolaise

**DOI:** 10.11604/pamj.2014.19.336.5038

**Published:** 2014-11-28

**Authors:** Gray A Wakamb Kanteng, Toni Kasole Lubala, Augustin Mulangu Mutombo, Gayllord Nkashama Mutoke, Aubin Ndjadi Kasongo, Stanis Okitotsho Wembonyama, Oscar Numbi Luboya

**Affiliations:** 1 Université de Lubumbashi, Faculté de Médecine, Département de Pédiatrie, Lubumbashi, République Démocratique du Congo

**Keywords:** Perception maternelle, allaitement, diversification alimentaire, Lubumbashi, maternal perception, breastfeeding, dietary diversification, Lubumbashi

## Abstract

**Introduction:**

Les habitudes d'allaitement et de diversification alimentaire sont sensiblement influencées par la perception des mères allaitantes. L'objectif de ce travail est d’évaluer la perception de ces femmes en milieu urbain à Lubumbashi (RD Congo) afin de relever les barrières à la conduite d'un allaitement optimal.

**Méthodes:**

Une étude qualitative transversale a été menée, basée sur des groupes de discussion menées dans 3 centres urbains de la ville de Lubumbashi, et concernant 76 mères avec leurs nourrissons.

**Résultats:**

Sur 76 mères, 52 n'ont pas pratiqué un allaitement maternel exclusif avant 6 mois. Les principales raisons évoquées sont: le bébé pleurait beaucoup (ne se rassasie pas), le bébé ne prenait pas suffisamment de poids, la production lactée insuffisante, le manque de temps, une interdiction médicale, des raisons de pudeur ou d'esthétique corporelle. Les personnes ayant conseillé le choix ou le moment de la diversification sont: la mère elle-même, un membre de la famille, un ami, le conjoint.

**Conclusion:**

La promotion d'un programme de santé visant à améliorer la perception des mères sur la pratique de l'allaitement maternel et de la diversification est un axe important pour améliorer le taux d'allaitement maternel exclusif avant 6 mois.

## Introduction

Depuis toujours, les habitudes alimentaires d'une population sont influencées par plusieurs facteurs exogènes, notamment socio-culturels et environnementaux. Plusieurs études attestent que ces considérations influencent la conduite de l'alimentation chez les nourrissons, parfois au détriment des recommandations consensuelles basées sur les évidences [1]. Au Vietnam et au Liban par exemple, des études révèlent que la publicité agressive sur les laits infantiles à laquelle les mères sont fort exposées en milieu urbain, le niveau économique plus favorable à l'achat des laits infantiles ainsi que l'accessibilité de ces produits dans le commerce favoriseraient l'abandon de l'allaitement maternel exclusif durant les 6 premiers mois [2, 3]. Ailleurs, en Afrique de l'Ouest, les facteurs culturels sont évoqués, où on note l'administration répandue et de manière précoce de liquides au nourrisson de sorte que le taux d'allaitement exclusif est globalement inférieur à 10% [4].

Dans d'autres séries Africaines, l'initiation précoce de l'allaitement est fréquente, et varie selon les milieux (zones urbaines et zones rurales). En Tanzanie par exemple, 82% des mères avaient initié l'allaitement dans la première heure de vie en milieu urbain contre 52% en milieu rural [5]. En 2010, le rapport MICS en RD Congo révélait que bien que l'allaitement soit quasi universel dans le pays (98%) et dans la province du Katanga en particulier (99%), seulement 38% de nouveau-nés ont été allaités dans l'heure qui a suivi la naissance. Par ailleurs, l’étude notait que 11% de nouveau-nés du Katanga ont reçu une nourriture pré lactée. Le taux d'allaitement exclusif jusqu’à 6 mois était de 31%. Et s'agissant de la poursuite de l'allaitement, les données montrent que la majorité des enfants (89%) âgés de 12-15 mois continuent d’être allaités et que cette proportion tombe à 42% chez les enfants âgés de 20-23 mois [6]. Cette étude qualitative a pour but de cerner auprès des mères congolaises vivant à Lubumbashi (province du Katanga), premières actrices en la matière, leur perception de l'allaitement au sein et de la diversification alimentaire. Ceci, pour relever les barrières à l'allaitement et envisager des pistes de solution.

## Méthodes

### Type d’étude

Nous avons procédé à une étude qualitative transversale afin de relever la perception des mères sur la pratique de l'allaitement maternel et de la diversification alimentaire. Pour ce faire il a été créé des groupes de discussion (focus group) avec usage d'un questionnaire d'interview pour guider les interviews.

### Choix du lieu d’étude

La présente étude a été menée en milieu urbain, dans la ville de Lubumbashi, au sud-est de la République Démocratique du Congo (dans la province du Katanga). Il s'agit de la deuxième ville la plus importante du pays, en termes de superficie et de démographie. Les groupes de discussions ont été organisés au sein des centres de protection maternelle et infantile (PMI) au moment où les mères apportent les nourrissons pour les consultations préscolaires. Ces centres de PMI sont intégrés à trois hôpitaux qui ont été choisis, notamment les Cliniques Universitaires de Lubumbashi, l'Hôpital Général de Référence Jason Sendwe ainsi que l'Hôpital de Référence Kampemba. Le choix des PMI nous a semblé opportun dans la mesure où la vocation des consultations préscolaires qui s'y déroulent est d'apporter, en dehors du programme de vaccination, des conseils d'ordre diététiques aux mères, notamment celles qui allaitent ou qui ont des enfants en âge de diversification.

### Participants

Nous avons procédé à un échantillonnage théorique afin de recruter les participants à l’étude parmi les mères fréquentant avec leurs nourrissons les consultations préscolaires. 76 mères ayant des nourrissons de plus de 6 mois (ayant déjà dépassé l’âge requis de la diversification) et moins de deux ans ont été retenues dans l’étude.

### Collecte des données

L'enquête était conduite sur base d'un questionnaire contenant des questions ouvertes et semi-ouvertes qui a été préalablement testé aux Cliniques Universitaires de Lubumbashi. Après le prétest, les amendements nécessaires ont été apportés dans la version finale utilisée pour la réalisation de l'enquête définitive. L'enquête a été effectuée durant les mois de mai et juin 2014. Chaque fois, un enquêteur préalablement formé a mené le groupe de discussion avec les mères. L'interview était menée soit en français ou en langue vernaculaire swahili selon le degré de compréhension de la mère.

### Analyse et interpretation des données

La transcription des groupes de discussion et des interviews a été faite selon la méthode d'analyse par induction thématiques [7]. Les thèmes récurrents émergents à partir des données brutes ont été identifiés et résumés sur des feuilles de calcul. Par la suite, les thèmes majeurs et mineurs ont été générés de manière à donner un aperçu des perceptions et des expériences de l′allaitement maternel et de la diversification alimentaire des femmes. Le processus d′entrevue a été arrêté lorsque la saturation des thèmes a été atteinte. Pour les définitions des concepts, nous avons adopté les définitions de l′OMS de l′allaitement maternel exclusif et de la diversification alimentaire [8].

### Considérations éthiques

L’étude a été effectuée sous le consentement verbal libre et éclairé des parents présents de chaque enfant, après une explication brève du but de notre étude. Le respect de la confidentialité des sujets de notre étude a été assuré. Le protocole du présent travail a été soumis à l'approbation au Département de Pédiatrie de la faculté de Médecine de l'Université de Lubumbashi.

## Résultats

Sur 76 mères interrogées, 52 n'ont pas effectué un allaitement maternel exclusif. A la question « quelles sont les raisons ayant motivé le non allaitement exclusif durant les 6 premiers mois », les mères ont évoqué les raisons principales suivantes:

### Le bébé pleurait beaucoup; il ne se rassasiait pas

Il s'agit de la raison la plus évoquée pour justifier le non-respect de la période des ¬6 mois requis pour l'alimentation exclusive au sein (17/52 mères). Voici quelques témoignages:

*« Je sais qu'il faut allaiter mon enfant jusqu’à 6 mois. J'ai essayé. Pourtant il pleurait tout le temps; il me fatiguait. J'ai commencé par ajouter des biberons supplémentaires par intervalle de ses tétées lorsqu'il a eu 2 mois. Puis, à 3 mois, j'ai commencé à alourdir ses biberons avec des cuillères de farines infantiles. A quatre mois, il prenait déjà une bouillie de céréales précuite. A 5 mois il était initié au plat familial. »*

*« Je ne dormais plus la nuit. Mon bébé réclamait de téter tout le temps. Plus il grandissait, plus il réclamait. Peu avant qu'il atteigne 3 mois, j'ai décidé de lui donner une bouillie à base de farine industrielle qu'on trouve sur le marché. Le jour où j'ai commencé, j'ai dormi toute la nuit sans qu'il pleure pour réclamer de téter. J'ai donc résolu de continuer. »*

### Le bébé ne prenait pas du poids

La conception populaire des mères est qu'un enfant en bonne santé doit être gros. 11 mères estimaient que leurs enfants n’étaient pas suffisamment gros, au point de décider de diversifier précocement leur alimentation.

*« J'ai expliqué au pédiatre que mon enfant ne prenait pas du poids. Il m'a garanti qu'il avait une bonne courbe staturo-pondérale. Ma voisine a un fils du même âge; lui, était si gros’ Elle lui donnait déjà de la bouillie de céréales. J'ai compris qu'il fallait que je fasse de même*. »

*« J'ai commencé par donner des vitamines à mon bébé pour qu'il grossisse. Ça n'a pas beaucoup marché. Je suis passé à l’étape supérieure en lui faisant gouter un peu de thé au matin, puis du Bukari (pate cuite de maïs). Il y a beaucoup de vitamines là-dedans’ »*

### La production lactée insuffisante

Il ressort que plusieurs mères éprouvaient le sentiment de n'avoir pas suffisamment de lait (9/52).

« J'ai déjà deux enfants. Au tour de celui-ci, mes seins se sont fatigués et ne produisent pas suffisamment. J’étais obligé d'adjoindre au lait maternel un supplément lacté artificiel avant 6 mois. » « Quand mon enfant tétait, il tire sur le sein puis il se fatigue. Je ne produis pas beaucoup de lait. »

« Dans notre famille, toutes mes s'urs et moi-même avons une faible production lactée. On m'a conseillé de boire en abondance une boisson locale fermentée qui stimulerait la montée laiteuse, mais cela n'a pas marché. »

### Le manque de temps d'allaitement pour cause d'occupations professionnelles

Une proportion de 6/52 mères ont accusé leurs occupations personnelles comme la cause de la diversification précoce.

*« Mon patron ne m'a accordé que trois semaines après la maternité pour reprendre le travail. J'ai dû engager une bonne et je lui ai montré comment préparer des biberons. J'allaite mon enfant le matin avant de partit et quand je rentre du travail, au soir. »*

*« Je suis vendeuse au marché. Je dois produire de l'argent pour que le bébé lui-même et ses autres frères survivent. Je suis tout le temps en mouvement, impossible d'emmener mon bébé. Sa s'ur ainée se charge de le garder et de lui préparer son repas en mon absence. »*

### Une restriction médicale à l'allaitement

Parmi les mères, 3 ont évoqué une interdiction médicale comme motif soit à l'arrêt total de l'alimentation au sein ou à la diversification précoce.

### Raison de pudeur ou d'esthétique corporelle

Une certaine tranche de femmes, souvent jeunes et de niveau social élevé, ont évoqué des raisons d'intégrité physique comme raison pour la diversification précoce, voire l'arrêt complet de l'allaitement au sein.

*« C'est mon premier enfant. J'ai un peu honte de l'allaiter en public. Pourtant il y a toujours des visiteurs à la maison. » « J'ai de drôles de sensations quand mon enfant tête au sein. Ça me gêne, surtout quand il y a du monde autour de moi. Je préfère alterner avec un biberon. »*

*« Mon mari aime que je garde des seins fermes. Ils seront fanés si j'allaite longtemps le bébé. »*

A la question « qui a conseillé le moment de la diversification et/ou le choix de l'aliment de complément au lait maternel », les mères ont donné les raisons ci-après:

### Elle-même

Le choix personnel était souvent motivé par une expérience ancienne (chez les multipares) ou par l'influence passive des connaissances diffuses (publicités de produits alimentaires infantiles par les médias, avoir vu faire par la plupart, etc.). Le manque d'informations rationnelles et fiables sur la question de l'alimentation du nourrisson semble en définitive être une influence importante au choix inopportun du moment de la diversification.

### Un membre de famille

Il s'agit souvent d'une personne plus âgée et respecté dans la famille, presque toujours une femme ayant elle-même l'expérience de plusieurs maternités. Sur la liste des personnes de la famille ayant encouragé le choix et le moment de la diversification figurent: la mère de l'allaitante, la belle-mère, une tante, une sœur.

### Une amie

Il s'agissait souvent d'une voisine ou d'une marraine, elles-mêmes ayant déjà allaité au moins une fois.

### Son conjoint

Le rôle du mari est clairement ressorti. Certains hommes encouragent le choix d'un aliment supplémentaire comme pour prouver leur réussite sociale et leur capacité à couvrir tous les besoins de leur famille. Certains diminuent l'attachement des mères à leurs enfants pour regagner cette attention pour eux.

### Un membre du corps médical

Il est paradoxal de constater chez certaines mères (8/52) que ce serait un membre du corps médical (infirmier, médecin,...) qui aurait conseillé le moment de la diversification précoce.

## Discussion

Comme le confirme les études antérieures [6, 9], le taux d'allaitement maternel exclusif reste faible dans notre milieu, notamment parmi les femmes ayant participé à notre enquête. Il ressort de la présente étude que les obstacles à l'allaitement maternel exclusif tels que décrits par les mères sont multiples. Soit elles estiment que le nourrisson est vorace et pleure tout le temps pour réclamer ses tétées; que celui-ci ne prend pas suffisamment du poids; que la production lactée est insuffisante; qu'elles manquent de temps pour allaiter à cause de leur profession; ou alors les mères obéissent à un interdit médical; ou elles s'imposent une certaine intégrité corporelle contre l'allaitement maternel. Ces expériences sont pour la plupart subjectives; elles ne sont pas basées sur les évidences scientifiques multiples faisant état de la suffisance du lait maternel durant les 6 premiers mois, sauf pour des situations exceptionnelles. Entre autre l′allaitement maternel exclusif et prolongé permet de réduire l′incidence des infections gastro intestinales et des eczémas atopiques dans la première année de vie [10, 11]. De plus, un allaitement exclusif et prolongé améliore de 5.9 l′échelle globale du quotient intellectuel [12].

Il est évident que la perception des mères est profondément influencée par des facteurs exogènes, à savoir l'influence de leur entourage (membres de famille, conjoint, amis, voire leur compréhension des injonctions médicales). En rapport avec les facteurs sous-jacents à la pratique de l'allaitement maternel, une étude Gambienne en milieu rural atteste que la pratique de l'allaitement maternel et de l'alimentation du nourrisson sont nettement influencées par les traditions culturelles, véhiculées par les plus vieux, hommes et femmes, les pères de famille y compris. Parallèlement, l'alimentation avec un lait infantile y est perçue comme un avancement positif vers la modernisation [13]. Ces constats sont superposables à notre milieu. D'où l'intérêt évident d'encourager des séances de vulgarisation des bienfaits de l'allaitement maternel et des modalités pratiques d'un allaitement maternel optimal au cours des séances de consultations prénatales et préscolaires, dans un langage compréhensible et persuasif.

## Conclusion

La modification de la perception de l'allaitement maternel auprès des mères est assurément un axe important pour améliorer le taux d'allaitement maternel exclusif à Lubumbashi. Elle permettrait également d'améliorer la conduite de la diversification alimentaire. A cet effet, l'intérêt d'une politique sanitaire structurée est important, afin de développer d'une part un programme efficace de promotion de l'allaitement maternel (par exemple la promotion des heures d'allaitement pour femmes travailleuses) et de codifier d'autre part l'accessibilité aux substituts du lait maternel.
